# Autumnal Diarrhœa

**Published:** 1899-08-12

**Authors:** 


### AUTUMNAL DIARRHŒA.

In a paper recently read by Dr. Hope, medical officer of health for Liverpool, the connection of autumnal diarrhœa with heat and with rainfall is well brought out. The association of this disease with prolonged high temperature has long been recognised, the rise of the four foot earth thermometer to about 56 degrees Fah. being empirically taken as the indication of the condition, whatever it may be, which leads to the occurrence of epidemic diarrhœa. Many hypotheses have been put forward as to the meaning of this fact, which was demonstrated by the late Dr. Ballard, but whatever the meaning may be it is certain that the occurrence of such earth temperatures must involve a considerable rise of surface temperature and a considerable permanence of such elevation. Now one of the most important factors of a rise of earth temperature is an absence of rain along with the presence of heat, for nothing is so effectual in cooling the ground in this climate as the evaporation of moisture deposited in the form of rain. Hence, even if we only regard absence of rainfall as contributing to rise of earth temperature, we see its importance in promoting the conditions which favour the occurrence of diarrhœa. But Dr. Hope attributes to it another role, and he points to the deprivation of the scavenging power of rain as one great cause of the efficacy of drought in promoting diarrhœa at times when the temperature which is necessary for the occurrence of certain forms of decomposition in articles of diet is present. Among other examples he gives two extreme years, 1891 with a wet summer in which 16·0 inches of rain fell between June and September, and with 203 deaths from zymotic diarrhœa during the third quarter; and 1895, with a dry summer in which only 7·7 inches of rain fell in the same months, and with 819 deaths from zymotic diarrhœa in the same quarter, and he goes on to say that the difference in rainfall in the two years means that upwards of 900 millions of gallons of water were distributed to the city in the warm season of low mortality which were absent in the warm season of high mortality. Heavy rains, he says, are invariably followed by immense benefit, and the close connection between the thorough washing of streets, footwalks, roofs, gutters, drains, and sewers, and the diminution of autumnal diarrhœa is very striking, the explanation being that in a fine and warm summer the decomposing dust and dirt and filth which are largely unavoidable in cities, unless removed by washing, give rise to a filth-laden atmosphere, a fact which no one can fail to appreciate who has noticed the foul smells which arise in warm weather from cab ranks, omnibus stations, &c. The moral of this is that the water supplies of cities for public cleansing purposes should be free and unstinted, and that the paving of streets, and especially of back streets, should be of an impermeable character, so as to admit of thorough and frequent washing. Not that public cleanliness is the only factor in the prevention of this disease; but it is easy to understand how great are the benefits which must result when Nature applies an additional 900 millions of water for cleansing purposes at the time they are most needed. Nature does well. Let us imitate her.

